# Supplementary material for: Intratumoral Adipocyte-High Breast Cancer Enrich for Metastatic and Inflammation-Related Pathways but Associated with Less Cancer Cell Proliferation
Source: Int J Mol Sci. 2020 Aug 11;21(16):5744. doi: 10.3390/ijms21165744 (PMC7461211; doi:10.3390/ijms21165744)
Supplement: Supplementary file 1 [file ijms-21-05744-s001.zip › Adipocyte_Supplementary File 20200807_v04.docx]

| **Genes used to define Adipocytes** |
| --- |
| ADH1B, ADIPOQ, ATP1A2, ATP5G3, C6, CILP, COL5A3, DBI, DLAT, ECHDC1, GPD1, HADHA, HP, LBP, PLIN1, PNPLA2, PPP1R1A, PPP2R1B, PTGER3, SLC25A6, TF |

**Supplementary Table S1:** The genes used to define adipocytes in xCell, the computational algorithm reported by Aran et al [1].

1. Aran, D.; Hu, Z.; Butte, A. J., xCell: digitally portraying the tissue cellular heterogeneity landscape. *Genome biology* **2017,** 18, (1), 220.


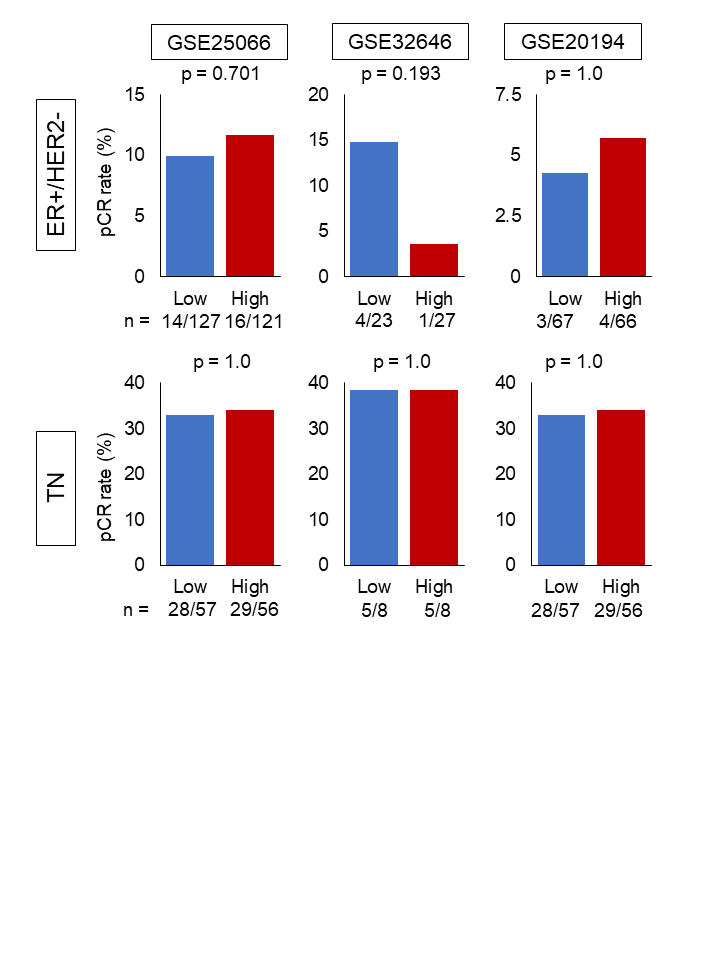


**Supplementary Figure S1.** Relationships between pathological complete response (pCR) rate to neoadjuvant chemotherapy (NAC) and amount of intratumoral adipocyte. Bar plots demonstrate the difference of pCR rate between the adipocyte-high (red) or low (blue) among the patients with ER+/HER2- or TNBC in GSE25066 (n = 508), GSE32646 (n = 115), and GSE20194 (n = 278) cohorts. Numbers of patients who were treated and those who achieved pCR are noted below the plots.

ER+/HER2-, ER positive and HER2 negative subtype; TN, triple negative subtype. p value < 0.05 was considered statistically significant.


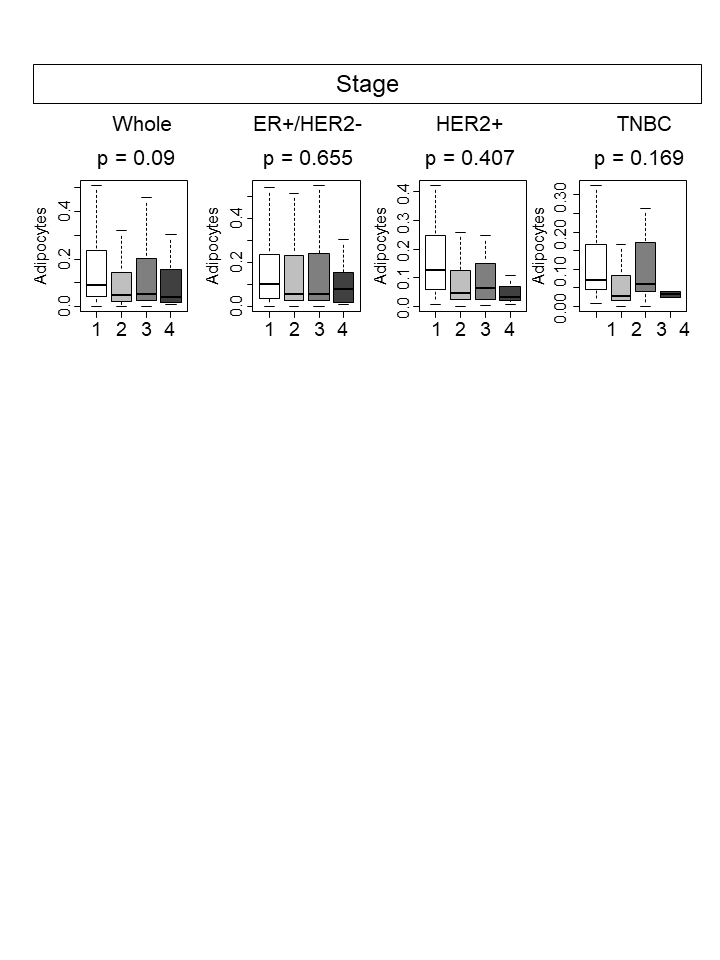


**Supplementary Figure S2.** Stage was not associated with the amount of adipocytes. p value < 0.05 was considered statistically significant.


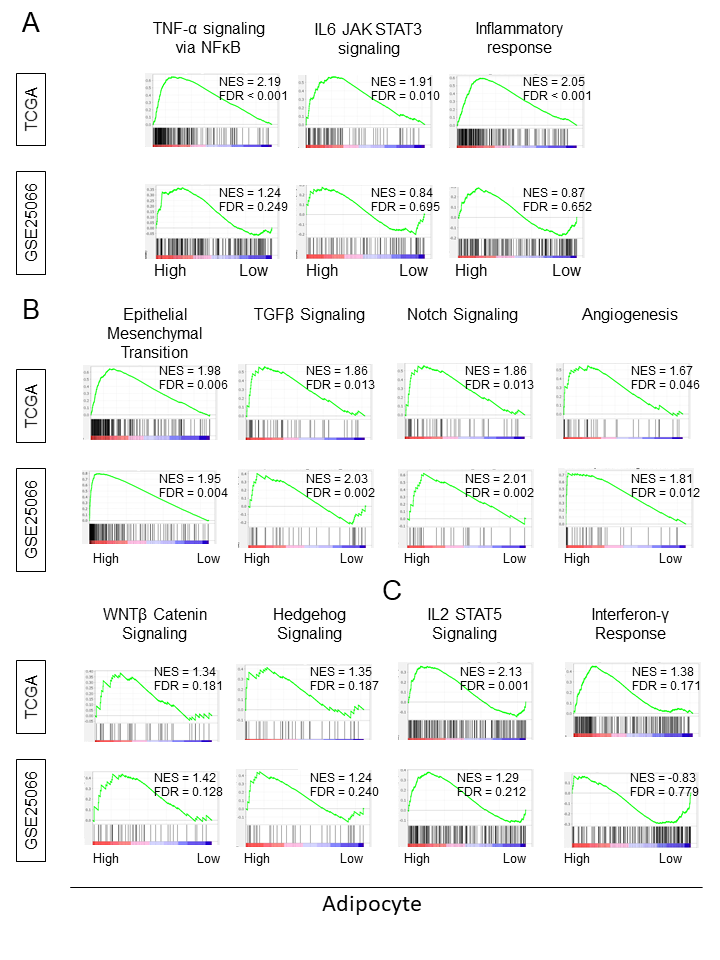


**Supplementary Figure S3.** Intratumoral-high adipocyte tumors enriched the gene sets related with inflammation (A), metastasis (B) and immune response (C) in ER+/HER2- subtype. ER+/HER2-, ER positive and HER2 negative. NES, normalized enrichment score; FDR, false discovery rate. FDR < 0.25 was considered statistically significant.


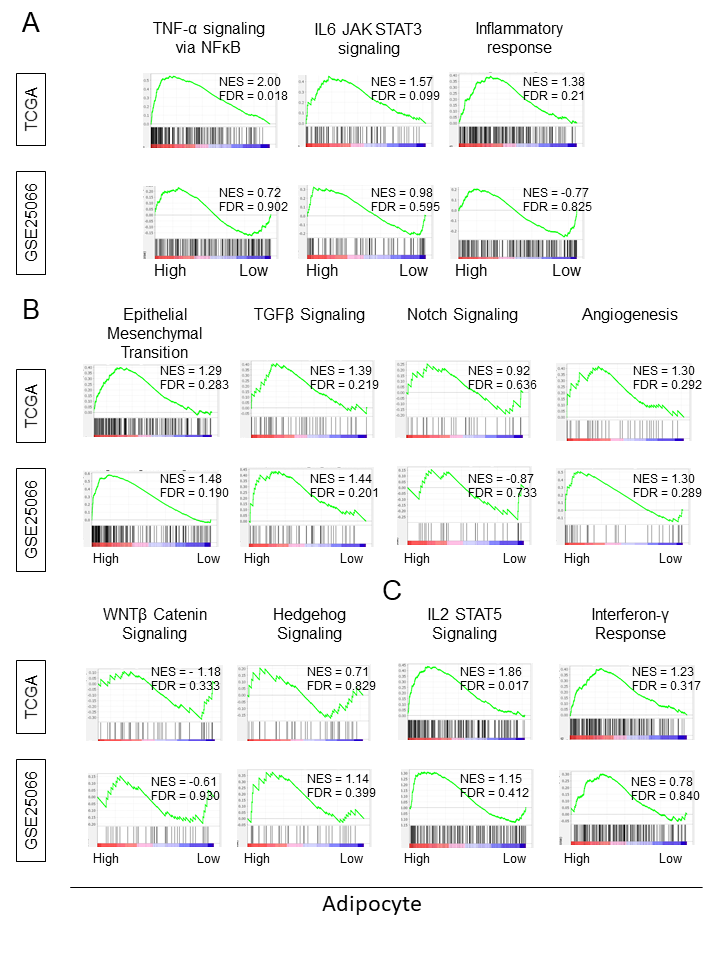


**Supplementary Figure S4.** Intratumoral-high adipocyte tumors are enriched for gene sets related with inflammation (A), metastasis (B) and immune response (C) in the TNBC subtype. TNBC, triple negative breast cancer. NES, normalized enrichment score; FDR, false discovery rate. FDR < 0.25 was considered statistically significant.


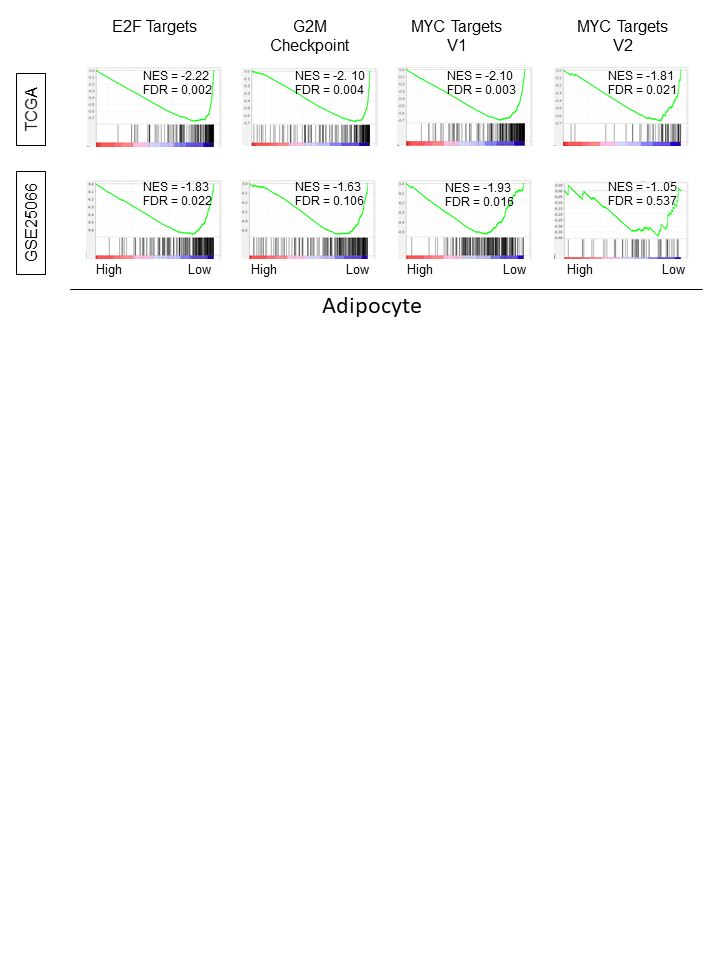


**Supplementary Figure S5.** GSEA demonstrated that the intratumoral adipocyte-low tumors enriched the gene sets associated with cell cycle and cell proliferation in ER+/HER2- subtype. ER+/HER2-, ER positive and HER2 negative. NES, normalized enrichment score; FDR, false discovery rate. FDR < 0.25 was considered statistically significant.


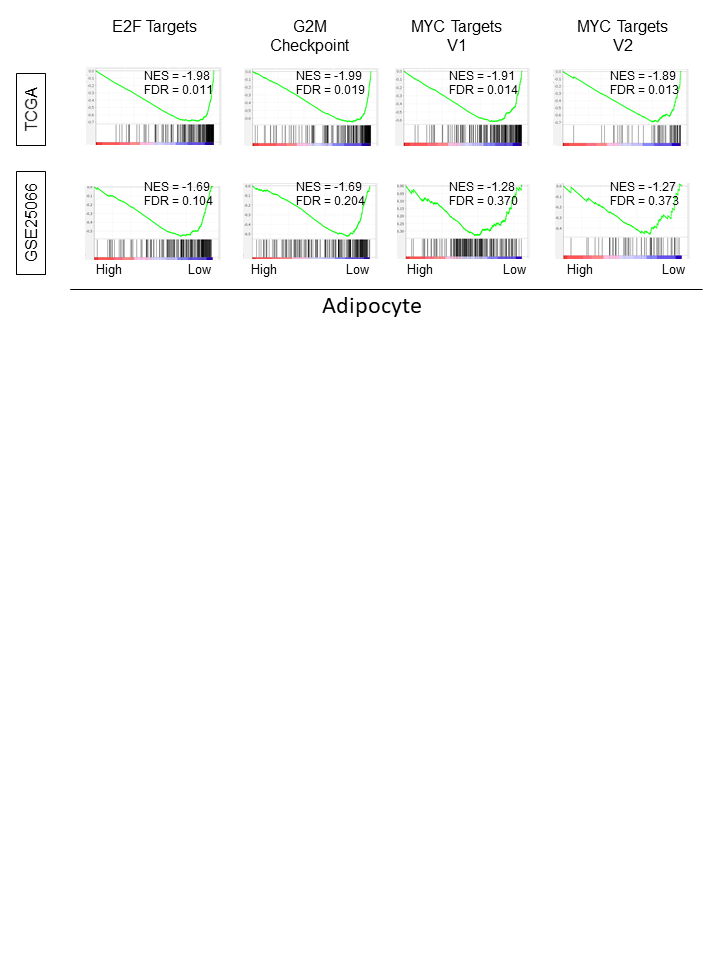


**Supplementary Figure S6.** GSEA demonstrated that the intratumoral adipocyte-low tumors enriched the gene sets associated with cell cycle and cell proliferation in TNBC subtype. TNBC, triple negative breast cancer. NES, normalized enrichment score; FDR, false discovery rate. FDR < 0.25 was considered statistically significant.
